# Huntington's Disease: A Report of an Interesting Case and Literature Review

**DOI:** 10.7759/cureus.55443

**Published:** 2024-03-03

**Authors:** Praveen K Sharma, Arun Aram, Yashaswinii Polaka, Vinoth Pandian

**Affiliations:** 1 Department of Radiology, Saveetha Medical College and Hospital, Saveetha Institute of Medical and Technical Sciences (SIMATS) Saveetha University, Chennai, IND; 2 Department of Radiology, Saveetha Medical college and Hospital, Saveetha Institute of Medical and Technical Sciences (SIMATS) Saveetha University, Chennai, IND

**Keywords:** magnetic resonance imaging, computed tomography, gabaergic neurons, basal ganglia, chorea, huntington disease

## Abstract

Huntington's disease (HD), referred to as Huntington's chorea, is an infrequent neurodegenerative ailment with an autosomal-dominant inheritance pattern characterized by the progressive deterioration of GABAergic neurons in the basal ganglia. Other ones include subcortical-type dementia, behavioral abnormalities, midlife psychosis, and gradual inadvertent choreoathetosis movements. HD is characterized by atrophy of the dorsal striatum (caudate nucleus and putamen) with concurrent expansion of the frontal horns of the lateral ventricles on imaging modalities such as computed tomography (CT) and magnetic resonance imaging (MRI). A molecular study validates the diagnosis of HD by identifying the disorder's hallmark amplified CAG triplet. Currently, there is no cure for HD, and treatment focuses on providing supportive care and managing the symptoms. Multidisciplinary approaches involving healthcare professionals, neurologists, and psychiatrists are crucial for comprehensive management. Medications are used to alleviate motor symptoms and manage psychiatric manifestations. Physical and occupational therapies help maintain functional abilities and improve quality of life. Genetic counseling and psychosocial support are essential for patients and their families. An additional crucial objective entails advancing more precise and dependable techniques for the timely identification and assessment of HD. Timely interventions and improved symptom management are made possible by early diagnosis. Based on clinical and imaging findings, we present a case of HD in a 62-year-old female.

## Introduction

Huntington's disease (HD) is a hereditary neurodegenerative disorder defined by the gradual degeneration of neurons inside the striatum, a component of the basal ganglia. The genetic pattern of HD follows an autosomal-dominant inheritance model. HD is characterized by symptoms affecting motor function, cognitive abilities, and mental well-being [1]. The genetic underpinnings of HD entail the occurrence of a CAG trinucleotide repeat expansion above 35-40 repeats on the huntingtin (HTT) gene located on chromosome 4 [2]. The correlation between the level of CAG expansion and the severity of HD has been identified, indicating that a greater number of repetitions accelerates the age at which symptoms first appear [3]. The frequency of HD exhibits variations across different ethnic groups, with a tendency toward higher rates among individuals of European descent. Reported rates have reached 10 to 15 cases per 100,000 individuals [4,5]. HD is distinguished by the presence of chorea, mental symptoms, and cognitive impairments [6,7].

## Case presentation

A 62-year-old female presented with unintentional, involuntary, irregular, non-patterned movements, behavioral changes, and progressive cognitive decline over the past three years. These movements were not associated with posturing. According to her husband's history, the patient had no complaints for three years and had been in good health until then. She has been experiencing uncontrolled jerking movements in her arms and legs, which have worsened over time. These movements are more noticeable during periods of stress or excitement and tend to improve during rest.

Additionally, he has observed that she has become increasingly forgetful, struggling with everyday tasks, and exhibiting emotional instability. There is no history of substance abuse or any significant past medical history. Her father was diagnosed at the age of 50 with HD. She has two siblings who are asymptomatic at present. No other significant neurological conditions are reported within the family. On physical examination, she appeared restless and demonstrated irregular, choreiform movements involving her face, trunk, and extremities. These movements are involuntary, purposeless, and involve rapid, jerky motions. She also displayed mild bradykinesia, slowed finger tapping, and diminished facial expressiveness. The observed movements exhibited a small range of motion, mostly impacting the extremities, facial region, and tongue. There was no associated imbalance or weakness of the limbs. Cognitive assessment reveals difficulties with attention, executive functions, and memory. However, no focal neurological deficits were noted.

**Figure 1:**
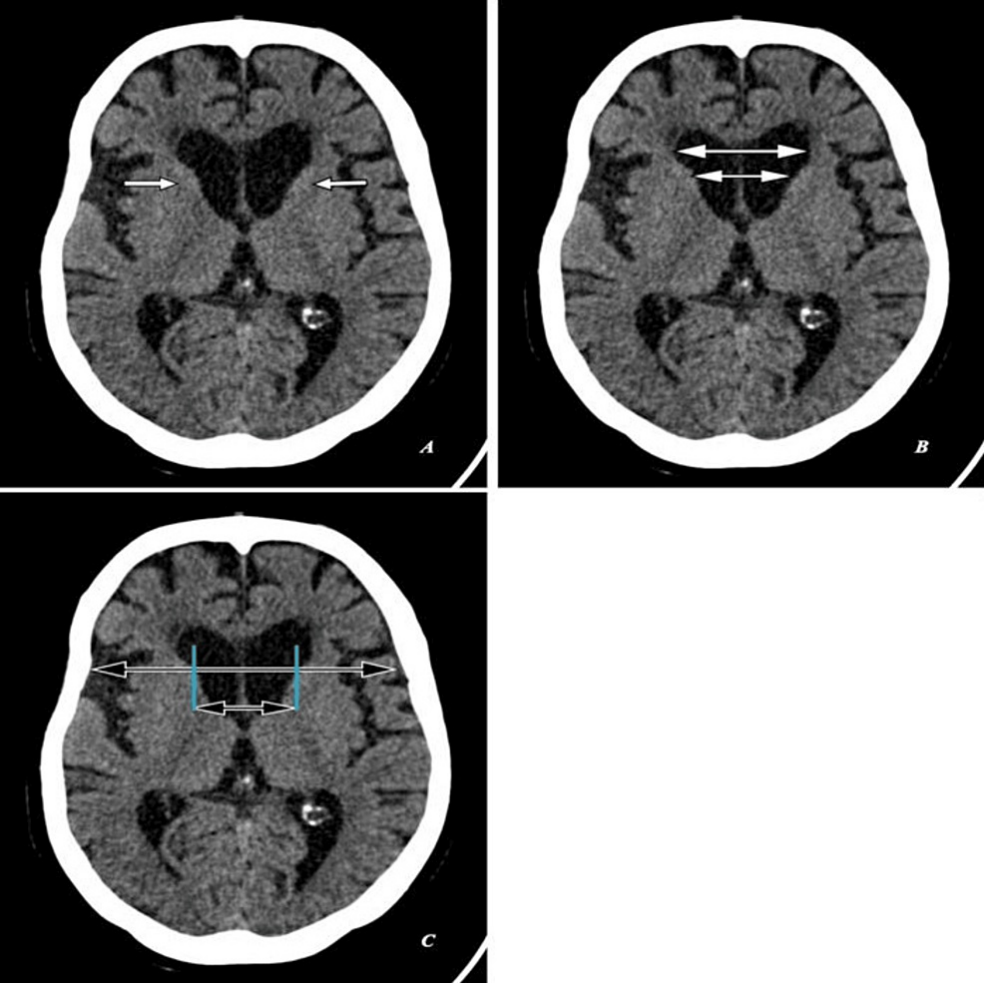
Computed tomography (CT) brain: (A,B,C) Axial sections of a 62-year-old female with unintentional, involuntary, irregular, non-patterned movements, behavioral changes, and progressive cognitive decline over the past three years. (A) Shows head of bilateral caudate nuclei mild atrophy with ex-vacuo mild dilatation of frontal horns of lateral ventricles (short white arrows). (B) Shows frontal horn width (large double white arrow) to intercaudate distance (small double white arrow) ratio (FH/CC) of 1.6, mildly decreased. (C) Shows an intercaudate distance (small double black arrow) to inner table width (large double black arrow) ratio (CC/IT) of 0.24, mildly increased.

All her laboratory investigations were unremarkable. Computed tomography (CT) brain showed head of bilateral caudate nuclei mild atrophy with ex-vacuo mild dilatation of frontal horns of lateral ventricles as seen in Figure 1A, with a frontal horn width to intercaudate distance ratio (FH/CC) of 1.6, mildly decreased as annotated in Figure 1B, and an intercaudate distance to inner table width ratio (CC/IT) of 0.24, mildly increased as annotated in Figure 1C. Associated with age-related diffuse brain parenchyma, mild atrophy with bilateral frontal CSF spaces, sulci, ventricular system, basal cisterns, posterior fossa CSF spaces, and cerebellar foliae appeared prominent (Figure 1A-1C).

**Figure 2:**
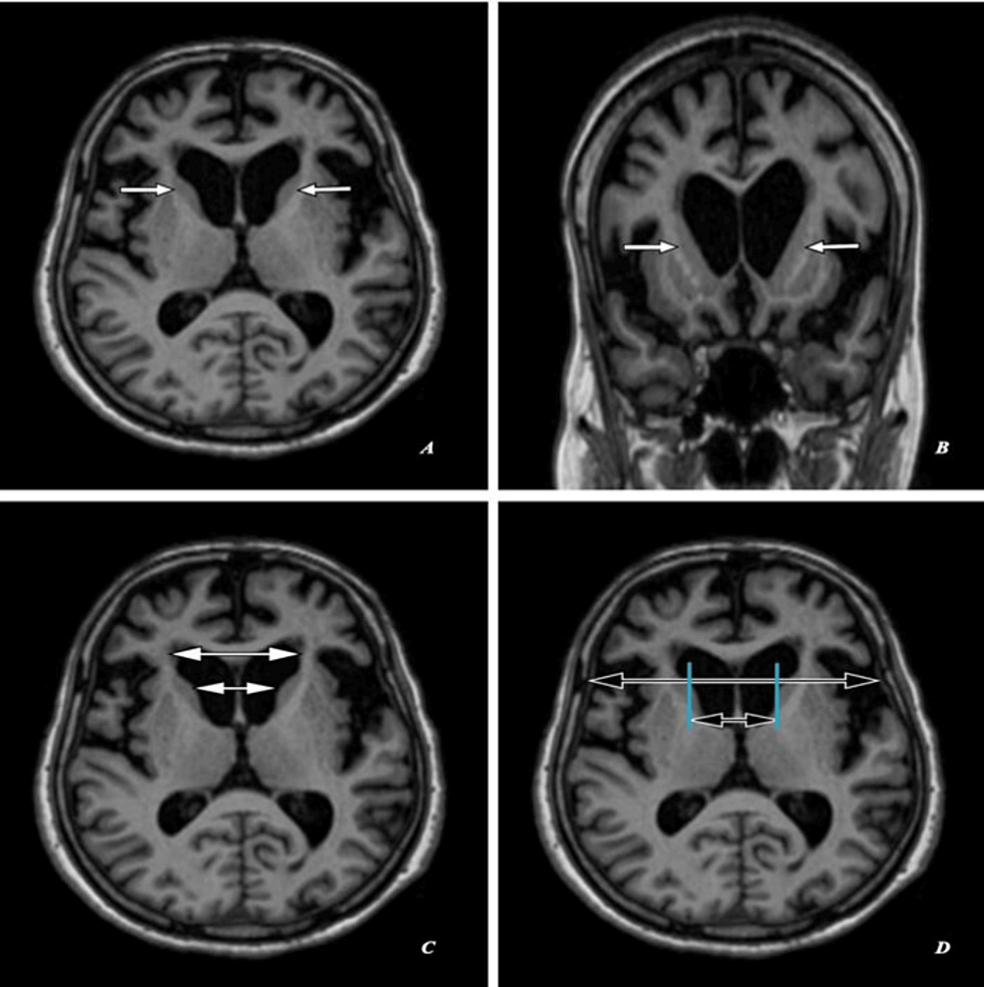
Magnetic resonance imaging (MRI) brain (T1-weighted): (A,C,D) Axial sections and (B) coronal section of the same patient. (A,B) Shows head of bilateral caudate nuclei mild atrophy with ex-vacuo mild dilatation of frontal horns of lateral ventricles (short white arrows). (C) Shows frontal horn width (large double white arrow) to intercaudate distance (small double white arrow) ratio (FH/CC) of 1.6, mildly decreased. (D) Shows an intercaudate distance (small double black arrow) to inner table width (large double black arrow) ratio (CC/IT) of 0.24, mildly increased.

**Figure 3:**
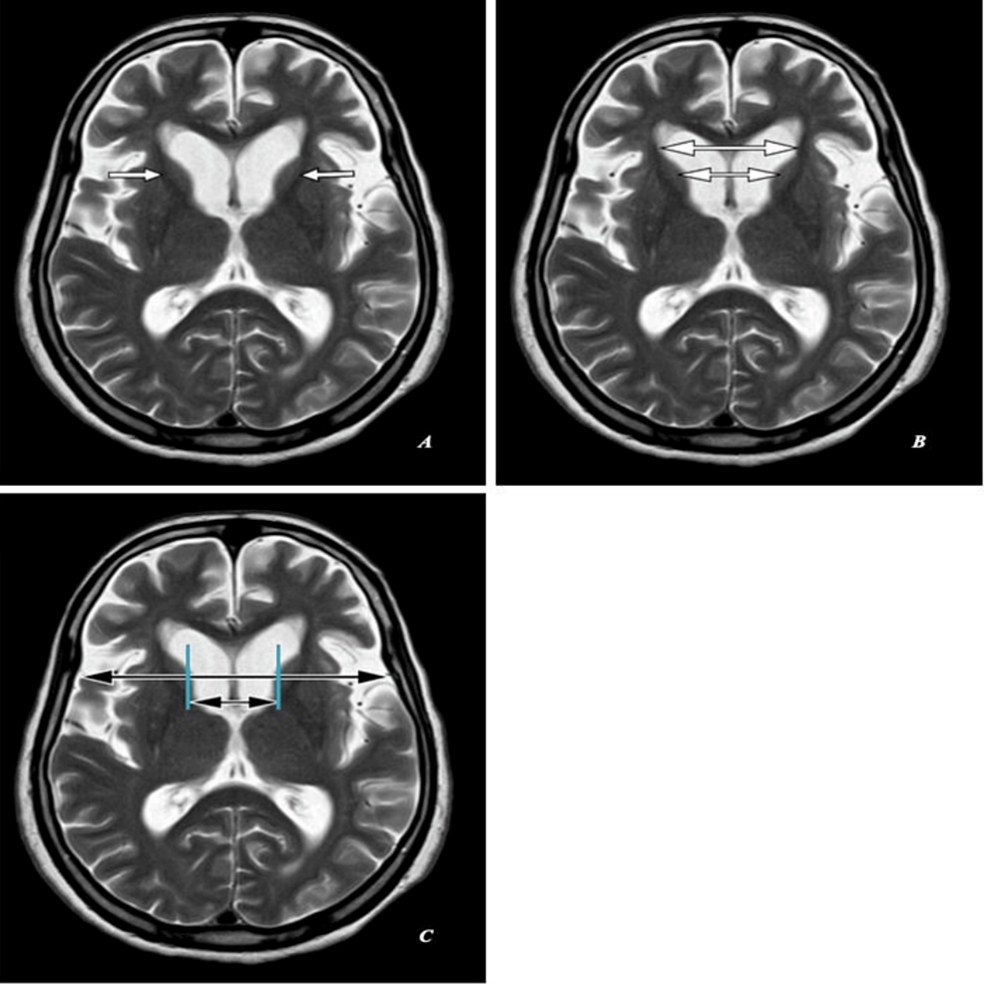
Magnetic resonance imaging (MRI) brain (T2-weighted): (A,B,C) Axial sections of the same patient. (A) Shows head of bilateral caudate nuclei mild atrophy with ex-vacuo mild dilatation of frontal horns of lateral ventricles (short white arrows). (B) Shows frontal horn width (large double white arrow) to intercaudate distance (small double white arrow) ratio (FH/CC) of 1.6, mildly decreased. (C) Shows an intercaudate distance (small double black arrow) to inner table width (large double black arrow) ratio (CC/IT) of 0.24, mildly increased.

**Figure 4:**
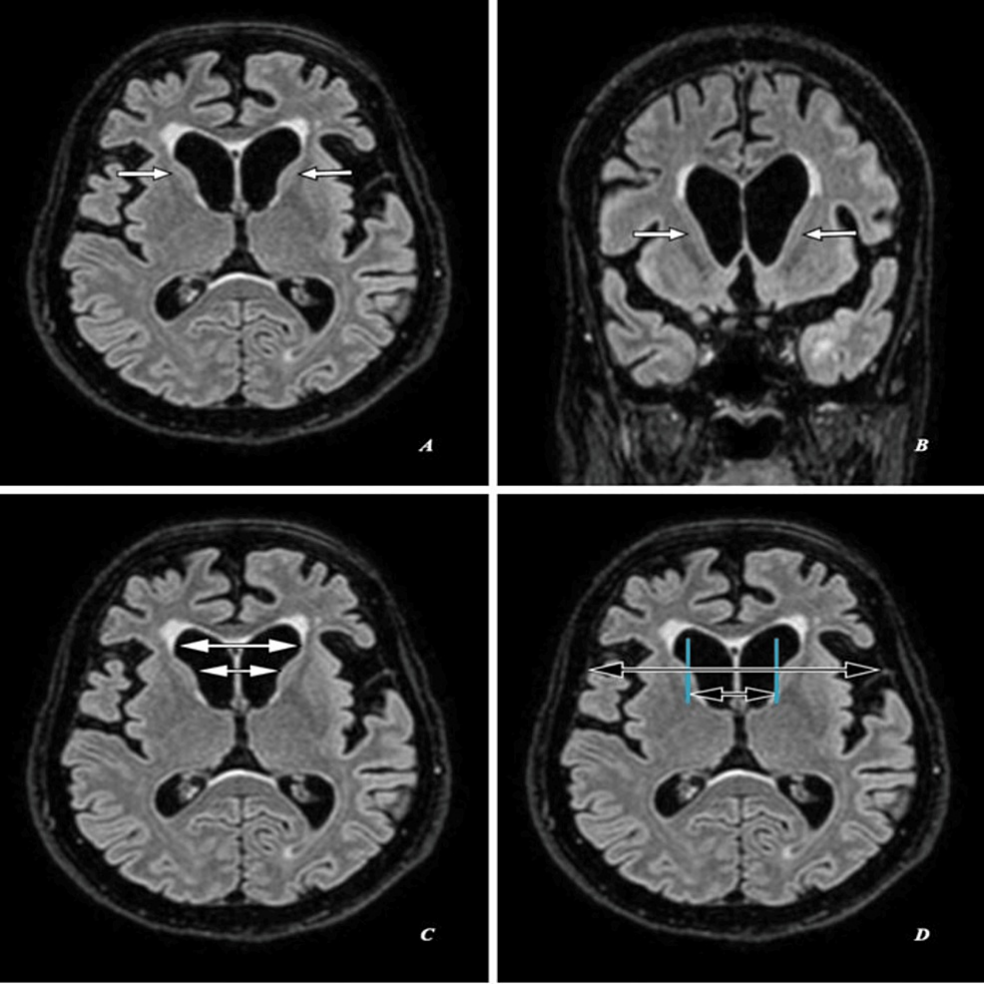
Magnetic resonance imaging (MRI) brain (fluid attenuation inversion recovery/FLAIR): (A,C,D) Axial sections and (B) coronal section of the same patient. (A,B) Shows head of bilateral caudate nuclei mild atrophy with ex-vacuo mild dilatation of frontal horns of lateral ventricles (short white arrows). (C) Shows frontal horn width (large double white arrow) to intercaudate distance (small double white arrow) ratio (FH/CC) of 1.6, mildly decreased. (D) Shows an intercaudate distance (small double black arrow) to inner table width (large double black arrow) ratio (CC/IT) of 0.24, mildly increased.

Magnetic resonance imaging (MRI) of the brain shows head of bilateral caudate nuclei mild atrophy with ex-vacuo mild dilatation of frontal horns of lateral ventricles as seen in Figures 2A, 3A, 4A with an FH/CC of 1.6, mildly decreased as annotated in Figures 2C, 3B, 4C and a CC/IT of 0.24, mildly increased as annotated in Figures 2D, 3C, 4D. Associated with age-related diffuse brain parenchyma, mild atrophy with bilateral frontal CSF spaces, sulci, ventricular system, basal cisterns, posterior fossa CSF spaces, and cerebellar foliae appeared prominent (Figures 2A-2D, 3A-3C, 4A-4D).

## Discussion

HD, also known as Huntington's chorea, is a rare autosomal-dominant trinucleotide repeat neurodegenerative disorder characterized by a loss of GABAergic neurons in the basal ganglia [1]. HD is a rare neurological ailment with an autosomal-dominant inheritance pattern, impacting approximately 1-7 individuals per million [1,2]. The condition has complete penetrance and is controlled by a gene on the p-arm of chromosome 4. The amplification of the trinucleotide causes HD CAG to repeat on chromosome 4. In the case of the juvenile variety, the disease is characterized by more than 60 repeats. HD is a neurological condition with an autosomal-dominant inheritance pattern, impacting approximately four to five individuals per million [8]. 

The clinical manifestation of HD is distinguished by a trio of symptoms encompassing motor impairments, cognitive decline, and psychological disturbances. Motor symptoms include chorea (involuntary jerky movements), dystonia (sustained muscle contractions), and various impairments in voluntary movements. Psychiatric manifestations, such as irritability, anxiety, and sadness, frequently precede the initiation of motor symptoms and are commonly observed. Others include rigidity (Westphal variant), dementia, and emotional disturbances [9,10].

HD presents with diverse clinical phenotypes, including choreatic, parkinsonian, mixed, psychiatric, cognitive, juvenile-onset, and late-onset phenotypes. The choreatic phenotype is characterized by involuntary jerky movements, while the Parkinsonian phenotype involves slowness of movement and rigidity. Mixed phenotypes combine choreatic and Parkinsonian features. Psychiatric symptoms such as depression and psychosis may precede motor symptoms in some cases, while cognitive impairment is common, particularly affecting executive functions and memory. Juvenile-onset HD manifests in childhood or adolescence with rapid progression, whereas late-onset cases occur later in life and maybe milder. Each phenotype requires tailored management approaches to address specific symptoms and needs.

The diagnostic process of HD involves the utilization of neuroimaging modalities, including computed tomography (CT) and MRI. The characteristic observations in HD consist of the degeneration of the basal ganglia (namely the corpus striatum), encompassing the caudate and putamen regions. This degeneration leads to the absence of the typical prominence in these structures. The caudate nucleus undergoes atrophy, leading to the absence of its characteristic bulge on the frontal horns, causing localized enlargement. HD progression is better appreciated on MRI than on CT and proceeds from dorsal to ventral and then medial to lateral. There will be atrophy of the caudate nucleus, leading to the absence of the typical bulge of these structures on the frontal horns, causing localized enlargement. Caudate nucleus atrophy and increased signal intensity on T2-weighted imaging in the basal ganglia and thalamus are common findings in HD [11,12]. Later in the disease progression, ventriculomegaly due to parenchymal atrophy becomes apparent. In the presented case, MRI revealed increased T2 signal throughout the caudate heads and lentiform nuclei, alongside mild ventriculomegaly. T2-weighted imaging may show either hyperintensity or hypointensity in the striatum, with hyperintensity attributed to neuronal loss and gliosis, and hypointensity to iron accumulation. While T2W hyperintensity is consistently reported in the rigid form of the disease, it is less common in the classical hyperkinetic form. MR spectroscopy often demonstrates reduced N-acetyl aspartate (NAA) and creatine levels in the basal ganglia, particularly in symptomatic patients. Advanced cases may exhibit involvement of other brain regions such as the pons, cerebellum, temporal lobes, and white matter tracts, with cortical atrophy correlating with neuropsychological symptoms. Additionally, functional MRI and diffusion tensor imaging (DTI) hold promise in identifying preclinical HD mutation carriers years before symptom onset [13,14].

Volumetric analyses indicate significant reductions in the volumes of striatal structures, with some decrease also noted in the thalamus and hippocampus. Research on mildly affected patients suggests a more pronounced atrophic change in the putamen compared to the caudate [13,14]. The ACPC (anterior commissure and posterior commissure) line, which measures the ratio between caudate heads and the inner table of the skull, is used to assess these structures. In this measurement, the normal range for the CC/IT ratio, taken from the frontal horns' caudate heads and lateral margins in the same axial plane, typically falls between 0.09 and 0.12. As the caudate heads shrink, the CC distance increases, leading to an increase in the CC/IT ratio, which typically averages between 2.2 and 2.6 for the FH/CC ratio [12].

DTI studies have revealed abnormalities in white matter integrity in HD. Fractional anisotropy values, which indicate the directionality of water diffusion, may be reduced in regions such as the corpus callosum and corticospinal tracts. These white matter changes contribute to disruptions in neural connectivity within the brain [13]. Both functional neuroimaging modalities, functional magnetic resonance imaging (fMRI) and positron emission tomography (PET), have exhibited modified patterns of cerebral activity in people diagnosed with HD. These imaging modalities reveal hypometabolism (reduced glucose metabolism) in the basal ganglia, frontal cortex, and other affected brain regions [13].

It is essential to understand that the radiological findings in HD can vary among individuals and throughout the disease. There is often a correlation between the degree and location of brain alterations and the clinical symptoms and course of diseases. Radiological assessment, combined with clinical evaluation, genetic testing, and other diagnostic measures, plays a crucial role in diagnosing and monitoring HD. Significant strides have been made in recent research, which has led to an understanding of the pathogenesis of HD and the identification of potential therapeutic targets. Strategies under investigation include gene silencing techniques, protein clearance approaches, and neuroprotective therapies. Clinical trials to evaluate the safety and efficacy of these novel interventions are underway, raising hopes for disease-modifying treatments in the future and holding promise for HD treatment [14].

Differentials include 1) Parkinson's disease; both are neurodegenerative disorders that can affect movement. However, the specific movement symptoms, progression, and other clinical features differ. 2) Wilson's illness is an inherited genetic ailment characterized by the excessive build-up of copper within the human body, resulting in detrimental effects on both the liver and the neurological system. The condition may manifest with motor impairments and psychological manifestations. Others may include spinocerebellar ataxias, dentatorubral-pallidoluysian atrophy, and metabolic disorders. Certain psychiatric conditions, such as bipolar disorder or schizophrenia, may initially present with psychiatric symptoms similar to those seen in HD [14].

One of the inherent limitations of a case report is the reliance on a single or very limited number of cases. This can restrict the generalizability of findings and may not adequately represent the heterogeneity of HD manifestations. The selection of a specific case for reporting may introduce bias, especially if the chosen case is not representative of the broader HD population. This can impact the applicability of findings to a more diverse range of individuals with the disease. Genetic counseling is crucial for individuals with a family history of HD. The case report underscores the importance of early genetic testing to identify carriers of the mutant gene. Healthcare professionals must be adept at providing information about the implications of HD, helping individuals make informed decisions about testing, and supporting them through the process.

HD affects various aspects of a person's life, including motor function, cognition, and mental health. A multidisciplinary care approach involving neurologists, psychiatrists, physical therapists, and social workers is essential. The case report emphasizes the need for healthcare teams to collaborate and tailor treatment plans to address the diverse challenges faced by individuals with HD. HD often has a familial component, and the impact on family members can be profound. Education and support for family members, both emotionally and practically, are critical. Learning points include the importance of involving family members in the care process, facilitating open communication, and addressing the psychological and social aspects of living with or caring for someone with HD. As HD is a genetic disorder, ethical considerations arise regarding predictive testing, family planning, and disclosure of genetic information. Healthcare professionals must navigate these complex ethical issues with sensitivity. The case report highlights the need for ongoing discussions about the ethical implications of HD, including the balance between autonomy and the potential impact on family dynamics. The case report serves as a reminder of the ongoing need for research into the pathophysiology of HD and the development of effective treatments. Healthcare professionals should stay informed about the latest research findings and potential therapeutic interventions, actively participating in or supporting research initiatives aimed at understanding and treating HD.

 Aylward et al. conducted MR studies indicating that both the frontal horn/caudate nucleus (FH/CC) and bicaudate (CC/IT) ratios closely correspond with caudate volume in individuals with HD [15]. Additionally, Mirowitz et al. observed bilateral symmetric basal ganglia T2 hyperintensity in two pediatric patients with HD among their series of 65 pediatric patients with neurodegenerative diseases [16]. Telenius et al. demonstrated increased lengths of CAG repeats and mosaicism not only in the brains but also in the sperm of HD patients [17]. Pavese et al. [18] utilized raclopride to exhibit decreased cortical binding in a significant portion of symptomatic HD subjects and premanifest carriers, particularly affecting the temporal and frontal regions. Those with reduced binding showed poorer performance in executive function and attention tests compared to those with normal cortical binding.

In contrast, Esmaeilzadeh et al.'s [19] PET study using [11C] FLB 457 found no significant alterations in D2 receptor binding compared to controls in the thalamus and cortex of early- to middle-stage HD subjects, although reductions were observed in the striatum as expected. Gamez et al. [20] employed [123I] FP-CIT, a ligand selective for the presynaptic dopamine transporter, to detect semiquantitative reductions in four out of 12 HD subjects, primarily affecting the putamen. More substantial reductions in ligand uptake were associated with worse Unified Huntington's Disease Rating Scale (UHDRS) scores.

## Conclusions

In conclusion, this case highlights a 62-year-old female who presented with HD, presenting classic clinical features. The patient was prescribed anti-psychotic medications to manage her psychiatric symptoms alongside which she was offered genetic counseling and cognitive support. She was also started on dopaminergic medications like levodopa and is on regular monitoring and follow-up. Her family was also counseled on the long-term care and were advised to provide emotional support to the patient. Multidisciplinary approaches involving psychiatrists, other healthcare professionals, and neurologists are crucial for comprehensive management.
